# European Society of Endocrine Surgeons (ESES) consensus statement on standardization and research requirements for intraoperative nerve monitoring in endocrine surgery

**DOI:** 10.1093/bjs/znag090

**Published:** 2026-08-06

**Authors:** Katrin Brauckhoff, Agata Dukaczewska, Pietro Princi, Rick Schneider, Sam Van Slycke, Selen S Yaliman, Julia I Staubitz-Vernazza, Neil S Tolley, Kyriakos Vamvakidis, Ilias A Zorbas, Thomas J Musholt

**Affiliations:** Department of Breast and Endocrine Surgery, Haukeland University Hospital, Bergen, Norway; Department of Clinical Science, University of Bergen, Bergen, Norway; Digital Health Cluster, Hasso-Plattner Institute, Potsdam, Germany; Multifunctional Centre of Endocrine Surgery, Cristo Re General Hospital, Rome, Italy; Departement of Medicine, Unicamillus International Medical University, Rome, Italy; Department of Visceral, Vascular, and Endocrine Surgery, Martin Luther University of Halle-Wittenberg, Halle (Saale), Germany; Department of General and Endocrine Surgery, AZORG, Aalst, Belgium; Department of General Surgery, Cerrahpasa Faculty of Medicine, Istanbul University-Cerrahpasa, Istanbul, Turkey; Section of Endocrine Surgery, Department of General, Visceral, and Transplantation Surgery, University Medical Centre of the Johannes Gutenberg University Mainz, Mainz, Germany; Department of Endocrine and Thyroid Surgery, Hammersmith Hospital, Imperial College NHS Healthcare Trust, London, UK; Department of Endocrine Surgery, Henry Dunant Hospital Centre, Athens, Greece; Department of Endocrine Surgery, Henry Dunant Hospital Centre, Athens, Greece; Section of Endocrine Surgery, Department of General, Visceral, and Transplantation Surgery, University Medical Centre of the Johannes Gutenberg University Mainz, Mainz, Germany

## Abstract

**Background:**

Intraoperative nerve monitoring (IONM) is widely used in endocrine surgery, yet substantial heterogeneity exists in its documentation, interpretation, and reporting. This limits comparability across studies and hampers the evaluation of clinical outcomes.

**Methods:**

A consensus process was conducted by the European Society of Endocrine Surgeons (ESES), combining a systematic literature review, analysis of Eurocrine^®^ registry data, a survey among ESES members, and a structured Delphi consensus process.

**Results:**

Considerable variability was identified in monitoring techniques, signal interpretation, and outcome reporting. Key areas requiring standardization include the definition of loss of signal, documentation of neuromonitoring parameters, reporting of technical settings, and objective assessment of vocal cord function. Based on these findings, a set of consensus statements was developed to define minimum requirements for IONM research and reporting.

**Conclusion:**

This consensus statement provides a structured framework to improve standardization, data quality, and comparability in IONM research. Adoption of the recommendations may facilitate more robust evaluation of clinical outcomes and support future evidence generation in endocrine surgery.

## Teaser

This European Society of Endocrine Surgeons (ESES) consensus statement addresses the heterogeneity in the documentation, interpretation, and reporting of intraoperative nerve monitoring (IONM) in endocrine neck surgery. Based on a systematic literature review, analysis of Eurocrine^®^ registry data, a survey among ESES members, and a structured Delphi consensus process, the consensus defines key standards for the methodological use and scientific evaluation of IONM. The resulting statements aim to improve the transparency, comparability, and quality of future IONM research.

## Introduction

Intraoperative nerve monitoring (IONM) has been used in endocrine neck surgery for several decades and is now widely integrated into routine clinical practice across Europe and beyond. Its use is supported by international guidelines and professional society statements, particularly in bilateral thyroid surgery and in procedures associated with an increased risk of nerve injury, such as redo surgery, large goitres, invasive thyroid malignancy, and anatomically complex cases^1–3^. In these settings, IONM is primarily intended to support intraoperative decision-making by enabling early detection of functional nerve impairment and facilitating changes in surgical strategy aimed at reducing the risk of bilateral vocal cord paralysis^4^.

Parallel to its broader clinical adoption, technical developments have expanded the scope of IONM, including different monitoring systems, different stimulation modalities, different recording techniques, as well as the introduction of continuous IONM^5,6^. While the fundamental principles of nerve stimulation and electromyographic signal recording are shared across systems, relevant differences exist in signal acquisition, display, documentation, and data storage. IONM has evolved from a selectively applied adjunct into a commonly used tool in contemporary endocrine neck surgery. This widespread implementation has also increased expectations regarding the role of IONM data in quality assurance and clinical research^7,8^.

Despite its long-standing use and broad clinical acceptance, the scientific literature on IONM remains heterogeneous. Previous reviews and guidelines have highlighted substantial variation in study design, data quality assessment, monitoring protocols, definitions of loss of signal (LOS), reporting of electromyographic parameters, and assessment of vocal cord function. This heterogeneity limits comparability between studies and complicates the interpretation of results.

Against this background, the European Society of Endocrine Surgeons (ESES) conducted a structured consensus process to address methodological variability in studies involving IONM. This position statement defines research requirements for the standardized collection, interpretation, and reporting of IONM data, with the aim of improving transparency and comparability across future studies.

At the 11th Biennial ESES Conference in Izmir, Turkey, in 2025, the proposed position statement was presented for discussion and voting. The consensus process was coordinated by an ESES working group, comprising the authors of the present manuscript.

## Methods

### Study design

This study was conducted as a consensus initiative of the ESES with the aim of evaluating current practice, identifying methodological limitations, and defining research requirements for studies involving IONM in thyroid surgery. A multimodal approach was applied, combining a systematic review of the literature, analysis of Eurocrine^®9^ registry data, a survey among ESES members, and a structured Delphi consensus process. The study was developed and coordinated by a designated ESES working group, with oversight and final approval by the ESES General Assembly (GA).

### Literature search and study selection

A systematic literature search was performed to identify studies reporting on the use of IONM in thyroid surgery. The search strategy was agreed upon by the working group and applied to three electronic databases: PubMed, Embase, and the Cochrane Library. The search query combined terms related to nerve monitoring and thyroid surgery as follows: (vagus nerve stimulation OR vagus nerve monitoring OR intraoperative neural monitoring) AND (thyroid OR thyroid surgery).

The search was conducted on 13 May 2024. All articles published from 2011 onwards were considered eligible, reflecting the publication date of the first representative international guideline by the International Intraoperative Monitoring Study Group (INMSG)^1^. Articles published before this date were excluded. After deduplication, titles and abstracts were screened independently by members of the working group using the Rayyan web-based tool^10^. Full-text review was performed for potentially eligible articles.

Studies were included if they reported original clinical data on the use of IONM in thyroid surgery. The following exclusion criteria were applied, based on predefined consensus within the working group: exclusion of case reports, animal studies, single-expert opinion papers, studies published before 2011, and studies without recurrent laryngeal nerve-related outcome data. Moreover, articles published in languages for which no native speaker was available within the group were excluded. Included languages were English, German, Polish, Spanish, Greek, Turkish, Dutch, French, Italian, Norwegian, and Swedish.

### Full-text analysis and data extraction

Full-text analysis was performed in small expert subgroups, each focusing on predefined aspects of IONM research quality and reporting. One subgroup evaluated adherence to established IONM standards and algorithms, including documentation of vagus and recurrent laryngeal nerve stimulation and assessment of postoperative vocal cord function (L1–V1–R1–R2–V2–L2). A second subgroup focused on technical aspects, including monitoring devices, stimulation modalities, electrode types, and methods of data recording and storage. A third subgroup assessed study design, methodological quality, and reporting of data quality. A formal methodological quality assessment was not applied, as the primary aim of the review was to evaluate reporting standards, technical transparency, and methodological heterogeneity rather than to estimate treatment effects.

### Registry data analysis

Eurocrine^®^ registry data for thyroid procedures performed between 1 January 2019 and 1 July 2024 were analysed. Use of anonymized Eurocrine^®^ registry data was approved by the Regional Committee for Medical and Health Research Ethics in Western Norway (approval number 748236). Extracted variables included patient demographics, use and type of IONM, documentation of nerve injury, change of surgical strategy after intraoperative nerve signal alterations, and performance of preoperative and postoperative laryngoscopy. Registry data were analysed descriptively.

### Survey among ESES members

An online survey was distributed to ESES members to assess current clinical practice and perceptions regarding the use, interpretation, and reliability of IONM. Survey items addressed indications for IONM, vagus nerve stimulation practices, interpretation of IONM signals, change of surgical strategy, and use of preoperative and postoperative laryngoscopy. Survey responses were analysed descriptively and used to inform the subsequent consensus process.

### Delphi consensus process and conference voting

A two-round Delphi consensus process was conducted using SurveyMonkey® software (SurveyMonkey Inc., San Mateo, CA, USA). In total, 61 experts from Europe with recognized experience in endocrine surgery and IONM were invited to participate. In the first round, participants rated proposed statements using a nine-point Likert scale ranging from 1 (strongly disagree) to 9 (strongly agree) and were given the opportunity to provide free-text comments. Statements were revised based on quantitative results and qualitative feedback before being redistributed in the second round. Consensus was predefined as a median score of 7–9 with an interquartile range (i.q.r.) ≤2 and at least 75% of responses within the 7–9 range. Outliers were defined as responses lying more than 1.5 × i.q.r. from the median. Statements that met the predefined criteria for consensus were retained as formal consensus statements.

Statements for which consensus was not reached during the Delphi consensus process, or which were considered controversial, were subsequently presented to all attendees at the 11th Biennial ESES Conference in Izmir, Turkey, in 2025,- hereafter referred to as the General Assembly (GA). These statements were discussed and put to an open vote among conference participants. The results of this voting process were recorded and are reported separately.

## Results

### Literature review and study characteristics

**Figure 1:**
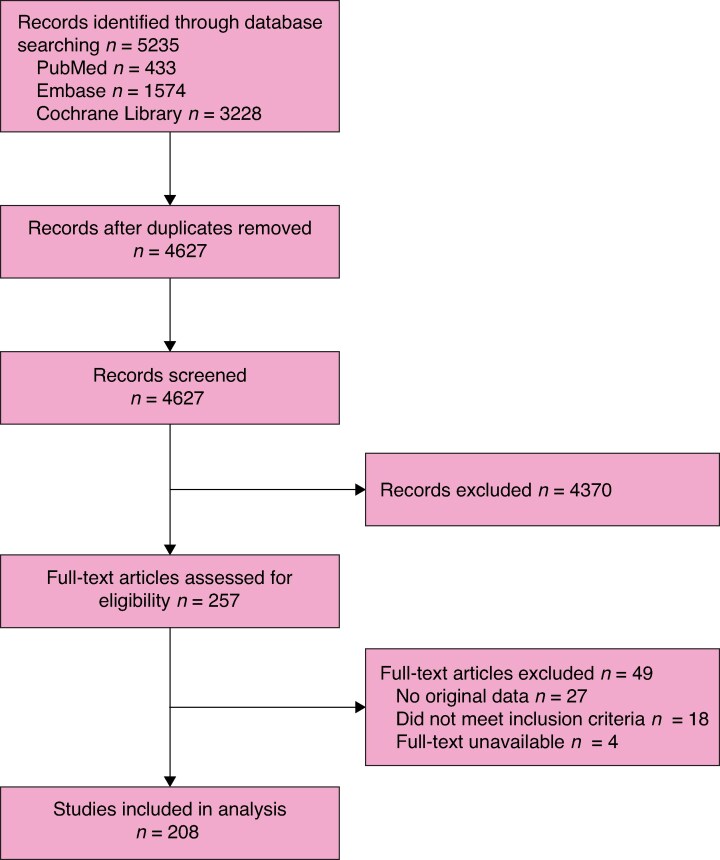
Flow diagram of study identification, screening, and selection Records were identified through database searching and screened according to predefined inclusion and exclusion criteria. Reasons for exclusion at the full-text stage are provided.

The systematic literature search identified a total of 5245 records across the three databases. After removal of duplicates and abstract screening, 208 studies were included in the final analysis (*Fig. 1*). The most common reasons for exclusion were inappropriate study design, inconsistent study population, lack of relevant outcome measures, and insufficient reporting of recurrent laryngeal nerve-related data.

Of the 208 studies, 89 studies (42.8%) were retrospective analyses, while 18 studies (8.7%) analysed prospectively collected data. Sixty studies (28.8%) were prospectively designed. The remaining 41 studies (19.7%) did not clearly specify whether data collection was retrospective or prospective. Most studies were monocentric (186 studies (89.4%)), with only 8 multicentric studies (3.8%) and 12 studies (5.8%) based on registry data.

Regarding study design, observational studies predominated (191 studies (91.8%)), including cohort studies (145 studies (69.7%)) and cross-sectional analyses (13 studies (6.3%)). Interventional studies were uncommon (13 studies (6.3%)) and only eight RCTs (3.9%) were identified.

### General use of IONM

**Statement 1**: IONM should be considered an essential tool for reducing the risk of recurrent laryngeal nerve injury in endocrine neck surgery.

Median 9 (i.q.r. 1)

Outliers 2

96% rated 7–9

**Strong Consensus**

**Statement 2**: The use of IONM improves surgical decision-making in cases with anatomical variations or distorted anatomy. IONM is particularly beneficial in bilateral thyroid surgery and in procedures associated with an increased risk of recurrent laryngeal nerve injury.

Median 9 (i.q.r. 1)

Outliers 1

98% rated 7–9

**Very Strong Consensus**

**Statement 3**: IONM is particularly beneficial in bilateral surgeries to prevent bilateral vocal cord paly and in high-risk cases, such as redo surgery, intrathoracic goitres, and invasive thyroid cancer. Therefore, IONM is strongly recommended in those interventions.

Median 9 (i.q.r. 0)

Outliers 0

100% rated 7–9

**Very Strong Consensus**

**Statement 4**: IONM should be considered a standard of care in endocrine neck surgery.

Median 8 (i.q.r. 1)

Outliers 4

92% rated 7–9

**Strong Consensus**

IONM is widely used in thyroid and endocrine neck surgery across many countries and healthcare systems, reflecting its broad clinical acceptance. Several professional society recommendations and guideline documents endorse the use of IONM^2,11,12^, particularly in higher-risk settings such as bilateral procedures, redo surgery, invasive malignancy, or when anatomy is distorted, both to support intraoperative decision-making and to help reduce the risk of bilateral vocal cord dysfunction. The systematic literature review identified 208 studies, representing 387 470 patients, highlighting the substantial global research interest in IONM and its widespread clinical use. In the Eurocrine^®^ registry (January 2019– July 2024), IONM was used in 80 484 of 92 505 thyroid procedures (87.0%), indicating that IONM is routinely implemented and, in many centres, effectively constitutes a standard component of thyroid surgery. Findings from the ESES member survey (161 ESES members) further reflected routine clinical use of IONM. Most respondents (84.5%) reported using IONM in more than 90% of thyroid procedures, with an additional 7.5% reporting use in more than half of cases, 4.3% reporting use sometimes (<50%), and only 3.7% reporting use rarely or never. In summary, the high prevalence of IONM use observed in both the Eurocrine^®^ registry data and the survey findings aligns with the strong to very strong consensus for Statements 1–4, supporting the view that IONM is regarded as a standard of care in contemporary thyroid surgery (*Table S1*).

### Application of the stimulation algorithm

**Statement 5**: Pre- and post-dissection vagus nerve stimulation is strongly recommended to establish an adequate IONM baseline signal and to reduce false-negative outcomes.

Median 9 (i.q.r. 1)

Outliers 1

98% rated 7–9

**Very Strong Consensus**

Survey data from ESES members demonstrated that pre- and post-dissection vagus nerve stimulation is widely practiced, but not uniformly applied. In the survey, 84.5% of respondents reported routinely stimulating the vagus nerve both before and after thyroid resection to assess nerve function. In a separate question, addressing the surgical approach, the frequency of vagus nerve exposure varied. Overall, 34.8% reported always exposing the vagus nerve, 21.1% reported often exposing the vagus nerve, and 29.2% reported sometimes exposing the vagus nerve. This variability reflects differences in surgical practice, case selection, and technical approach *(Table1)*.

Findings from the systematic literature review support this heterogeneity. Among 208 included studies, representing 387 470 patients, explicit exposure of the vagus nerve was reported in 154 studies (74.1%), while 14 studies (6.7%) reported no exposure and 40 studies (19.2%) did not report wether vagus nerve exposure was performed. Although the International Neuromonitoring Study Group algorithm (L1–V1–R1–R2–V2–L2) was described in 165 studies, full and complete application and reporting of the algorithm was documented for only 26.2% of patients.

Together, survey and literature data highlight that, although vagus nerve stimulation is recognized as a critical component of IONM, its implementation remains variable and is not yet fully standardized in clinical practice and research reporting. These findings directly support the very strong consensus of Statement 5, emphasizing the importance of systematic pre- and post-dissection vagus nerve stimulation to establish a reliable baseline signal and to reduce false-negative IONM results (*Table S1*).

### Technical aspects of IONM systems

**Statement 6**: Different nerve monitoring systems provide equivalent functionality in IONM, but surgeons should be aware of varying data storage and documentation methods, which may impact further evaluation, quality assurance, or research.

Median 8 (i.q.r. 1)

Outliers 3

94% rated 7–9

**Strong Consensus**

**Statement 7**: There is no significant difference in the effectiveness (or harm) of IONM when using a 1 *versus* 2 mA stimulation current, but awareness of stimulation settings (necessity of supramaximal stimulation) remains important for consistent interpretation of nerve signals.

Median 8 (i.q.r. 1.25)

Outliers 4

85% rated 7–9

**Strong Consensus**

**Statement 8**: The type of recording electrodes may influence signal quality and interpretation, impacting intraoperative decision-making and postoperative outcomes. Surgeons should be aware of differences in electrode design and placement techniques to ensure safe and practical use of IONM.

Median 8 (i.q.r. 1)

Outliers 1

98% rated 7–9

**Strong Consensus**

Across the 208 included studies, representing 387 470 patients, a variety of commercially available IONM systems were used, with no evidence that any specific platform was associated with superior intraoperative nerve identification or clinical performance. Systems from Medtronic (Minneapolis, MN, USA) were reported most frequently (65.2%), followed by Inomed (Emmendingen, Germany) (6.3%), Dr. Langer Medical (Waldkirch, Germany) (5.4%), and Neurosign (Magstim Company Ltd, Whitland, UK) (3.0%). Other devices or dual monitoring systems were used in 6.2% of studies. The monitoring system was not specified in 13.9% of studies (*Table 1*).

**Table 1:** Reporting of IONM systems, modalities, technical parameters, and data acquisition across included studies (*n* = 208)

	Values
**Device used**	
Medtronic	136 (65.2)
Inomed	13 (6.3)
Dr. Langer Medical	11 (5.4)
Neurosign	6 (3.0)
Double system	9 (4.3)
Other devices	4 (1.9)
Not reported	29 (13.9)
**Monitoring modality**	
Intermittent IONM	145 (69.7)
Continuous IONM	15 (7.2)
Both modalities	32 (15.4)
Not reported	16 (7.7)
**Data collection method**	
Institutional database	66 (31.7)
Snapshot recording	20 (9.6)
Printed output	6 (2.9)
Not specified	116 (55.8)
**Stimulation intensity**	
Reported	134 (64.4)
Not reported	74 (35.6)
**Vagus nerve exposure**	
Exposed	154 (74.1)
Not exposed	14 (6.7)
Not reported	40 (19.2)
**Recording electrode**	
Integrated endotracheal surface electrodes	128 (61.5)
Needle electrodes	12 (5.8)
Both surface and needle electrodes	13 (6.3)
Adhesive skin electrodes	1 (0.5)
Not specified	54 (26.0)

Values are *n* (%). IONM, intraoperative nerve monitoring.

Technical characteristics are summarized to illustrate variability relevant to data acquisition, documentation, and research comparability rather than to compare device performance (*Table 2*). Across the included studies, commercially available IONM systems differed with respect to stimulation parameters, configuration of stimulating probes and recording electrodes, signal display, and data storage options. While core functions, nerve stimulation and real-time electromyography (EMG) acquisition, were comparable, systems varied in their ability to document absolute amplitude and latency values, store continuous monitoring trends, and export data for further analysis. These differences directly influence auditability and the suitability of IONM data for research purposes. Detailed technical characteristics are summarized in *Table S2*.

**Table 2:** Technical characteristics of commonly used IONM systems

Parameter	Dr. Langer Medical (Avalanche^®^ SI2)	Inomed (C2 Xplore^®^)	Medtronic (NIM Vital™)
**System characteristics**			
Operating system	Windows^®^ 10	Windows^®^ 10	Windows^®^ 10
Stimulation type	Constant current	Constant current	Constant current
Current range (mA)	0.05–10	0.01–25	0.01–50
Frequency range (Hz)	3–4	0.1–60	1–20
Integrated data storage	Yes	Yes	No
HL7 integration	Yes	Yes	No
USB connectivity	Yes	Yes	Yes
**Stimulation devices**			
Intermittent monitoring (monopolar/bipolar)	Yes	Yes	Yes
Surgeon-controlled probe	No	No	Yes
C-IONM electrode type	Saxophone	Delta	APS
C-IONM configuration	Monopolar/bipolar	Bipolar	Monopolar
Default stimulation settings			
Intermittent frequency (Hz)	4	3	4
Pulse width (µs)	200	200	100
Current (mA)	1	1	1
Continuous frequency (Hz)	3	1	1
**Recording characteristics**			
EMG tube	Adhesive/flexible	Standard/adhesive	Integrated tube
Number of channels	1	4	1–2
Needle electrodes	Yes	Yes	Yes
EMG analysis			
Amplitude	Peak-to-peak	Peak-to-peak	Peak-to-peak
Latency definition	Onset latency	Onset latency	Onset latency
**Data display and monitoring**			
EMG event display	Yes	Yes	Yes
Continuous trend display	Yes	Yes	Yes
Threshold settings (latency/amplitude)	Adjustable	Adjustable	Adjustable
**Data storage and export**			
Snapshot storage	Yes	Yes	Yes
Continuous EMG storage	Yes	Yes	No
Raw data export	Yes	Yes	Yes

Comparison of key system features, stimulation settings, recording characteristics, and data-handling capabilities across commonly used IONM platforms. Detailed technical specifications are provided in *Table S2*. IONM, intraoperative nerve monitoring; HL7, health level seven; USB, universal serial bus; C-IONM, continuous intraoperative nerve monitoring; APS, automatic periodic stimulation; EMG, electromyography.

In the literature, EMG data were available for 78 studies (37.5%), with explicit EMG amplitudes reported in 72 studies (34.6%) and latency values in only 27 studies (13.0%). The stimulation intensity was documented in 134 studies (64.4%). Data collection methods were inconsistently described; institutional databases were used in 31.7% of studies, while more than half (55.8%) did not specify how IONM data were recorded or stored. Snapshot recordings (selection of a single ‘optimal’ EMG signal from a stimulation sequence and its labelling) (20 studies) and printed outputs (6 studies) were used infrequently and only four studies (1.9%) reported formal validation of IONM data before analysis.

Survey data from 161 ESES members corroborated these findings. While 40.4% of respondents reported that IONM curves from snapshot recordings were routinely stored in the patient record, 31.7% indicated that data were stored only on the monitoring device and 28.0% relied on external storage solutions.

Collectively, evidence from the literature, registry data, and survey responses demonstrate that, while contemporary IONM systems provide equivalent core functionality, heterogeneity in stimulation settings, characteristics of stimulating and recording electrodes, and data storage and documentation practices remains substantial. These differences may influence auditability, research usability, and inter-institutional comparability. Against this background, the working group reached strong or very strong consensus (Statements 6–8) that surgeons should be aware of system-specific documentation and data-handling characteristics to support quality assurance and reproducible research (*Table S1*).

### Preoperative and postoperative laryngoscopy

**Statement 9**: Preoperative laryngoscopy is strongly recommended in cases where there is a history of voice changes, prior neck or chest surgery, suspected malignancy with possible nerve involvement, or other risk factors for vocal cord dysfunction.

Median 9 (i.q.r. 1)

Outliers 6

88% rated 7–9

**Strong Consensus with some disagreement**

**GA voting: Agree 98%**

**Statement 10**: Routine preoperative laryngoscopy is recommended for all patients as vocal cord palsy can be asymptomatic. In research settings, it is essential to objectively document baseline vocal cord function. This ensures accurate assessment of nerve function outcomes and strengthens the validity of studies evaluating IONM or surgical technique.

Median 9 (i.q.r. 2)

Outliers 5

81% rated 7–9

**Moderate Consensus**

**GA voting: Agree 81%**

**Statement 11**: Postoperative laryngoscopy should be performed in patients with new-onset voice changes, swallowing difficulties, or when IONM indicates nerve injury.

Median 9 (i.q.r. 1)

Outliers 8

83% rated 7–9

**No Consensus**

**New Statement 11**: Postoperative laryngoscopy should be **at least** performed in patients with new-onset voice changes, swallowing difficulties, or when IONM indicates nerve injury.

**GA voting: Agreed 97%**

**Statement 12**: Routine postoperative laryngoscopy is essential in research settings to objectively document vocal cord function. This ensures accurate assessment of nerve function outcomes and strengthens the validity of studies evaluating IONM or surgical technique.

Median 9 (i.q.r. 1)

Outliers 5

90% rated 7–9

**Strong Consensus**

**Statement 13**: Although sensitivity and specificity of IONM performed with standard protocols in experienced hands is high, false-positive and false-negative results cannot be excluded completely. Therefore, preoperative and postoperative laryngoscopy remains the ‘gold standard’ for the assessment of vocal cord function.

Median 9 (i.q.r. 1)

Outliers 3

94% rated 7–9

**Strong Consensus**

Across the literature, registry data, and survey responses, substantial heterogeneity was observed regarding the use of preoperative and postoperative laryngoscopy, reflecting differences between risk-adapted clinical practice and routine baseline assessment, with many studies lacking standardized postoperative vocal cord assessment despite reporting nerve injury outcomes. In total, 18 studies, representing 274 695 patients (70.9% of all patients), did not report any laryngoscopic assessment at all. Among the remaining 190 studies (representing 112 758 patients), laryngoscopy was performed before surgery and after surgery in 168 studies, representing 101 101 patients (89.6%), whereas postoperative laryngoscopy alone was reported in 22 studies, representing 11 657 patients (10.4%).

**Figure 2:**
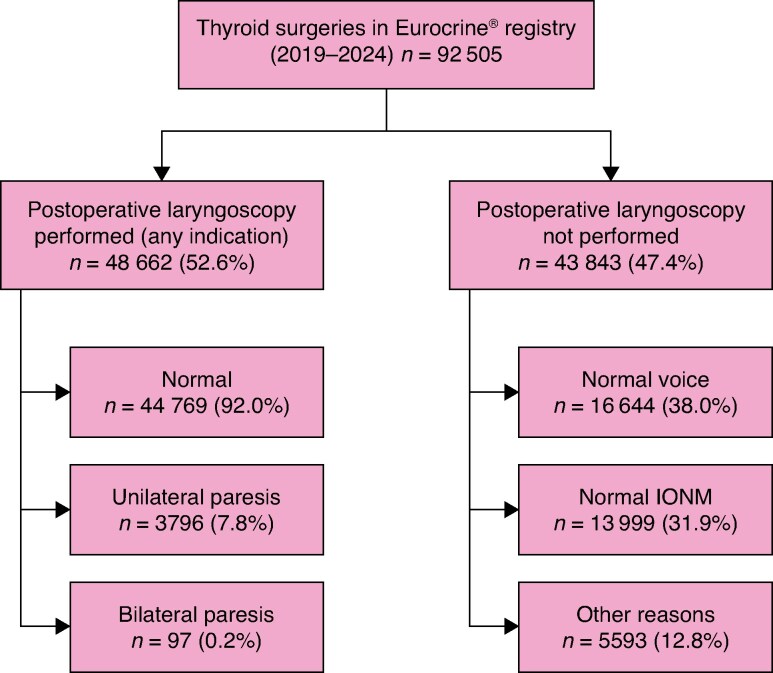
**Postoperative laryngoscopy in the Eurocrine^®^ registry (January 2019–July 2024).** Overall use of postoperative laryngoscopy following thyroid surgery and corresponding outcomes. Laryngoscopy performed includes examinations undertaken for any indication. For procedures without postoperative laryngoscopy, the documented reason was classified as normal voice, normal intraoperative nervemonitorering (IONM), or other. For some surgeries, no reason for omission was recorded in the registry.

Eurocrine^®^ registry data showed that preoperative laryngoscopy was documented in 68 707 of 92 505 thyroid procedures (74.0%). Pre-existing unilateral recurrent laryngeal nerve palsy was identified in 928 patients (1.4%) and bilateral palsy was identified in 29 patients, underlining the clinical relevance of baseline vocal cord assessment, particularly in patients with risk factors for nerve dysfunction. Routine postoperative laryngoscopy was documented in only 33 951 of 92 505 procedures (38.0%), indicating that postoperative vocal cord assessment is often performed selectively rather than systematically in routine clinical practice. *Figure 2* summarizes the use ogf postoperative laryngoscopy in the Eurocrine^®^ registry, including outcomes among patiens underwent laryngoscopy and documented reasons for omission when postoperative laryngoscopy was not performed. Survey responses from ESES members mirrored this pattern. Routine postoperative laryngoscopy in all patients was reported by 39.9% of respondents, while 33.1% performed postoperative laryngoscopy only after loss of IONM signal and 25.2% only performed postoperative laryngoscopy when clinical symptoms suggested vocal cord dysfunction.

Consistent with these findings, the ESES consensus demonstrated broad agreement on the importance of selective preoperative laryngoscopy. Strong consensus was reached for recommending preoperative laryngoscopy in patients with voice changes, prior neck or chest surgery, suspected malignancy with possible nerve involvement, or other risk factors for vocal cord dysfunction (Statement 9). In contrast, routine preoperative laryngoscopy in all patients (Statement 10) achieved only moderate consensus, reflecting ongoing variability in clinical practice, resource availability, and perceived cost–benefit outside research settings.

Variation in postoperative laryngoscopy practice corresponds to the lack of consensus observed for selective postoperative laryngoscopy based solely on symptoms or intraoperative findings (Statement 11). In contrast, strong consensus was achieved for the role of postoperative laryngoscopy in research settings to objectively document vocal cord function (Statement 12). Furthermore, despite high sensitivity and specificity of IONM when performed with standardized protocols in experienced hands, strong consensus was reached that preoperative and postoperative laryngoscopy remains the ‘gold standard’ for the assessment of vocal cord function (Statement 13). This reflects broad agreement that false-positive and false-negative IONM results cannot be completely excluded and that objective laryngoscopic evaluation remains indispensable for outcome validation. At the GA, voting confirmed broad support for the laryngoscopy-related statements. Risk-adapted preoperative laryngoscopy (Statement 9) was unanimously endorsed (98% agree; 153/156 participants), whereas routine preoperative laryngoscopy in all patients (Statement 10) attracted lower support (81% agree; 162/200 participants), indicating ongoing variation in clinical practice and perceived feasibility. Selective postoperative laryngoscopy based on symptoms and/or intraoperative IONM findings and an ‘at least performed’ wording (Statement 11) received 97.0% agreement (144/148 participants) (*Table S1*).

### Predictive value of IONM and signal interpretation

**Statement 14**: The presence of stable IONM signals throughout the procedure correlates strongly with postoperative vocal cord function preservation.

Median 8 (i.q.r. 1)

Outliers 3

94% rated 7–9

**Strong Consensus**

**Statement 15**: The predictive accuracy of IONM for postoperative vocal cord function is influenced by multiple factors, including institutional protocols, variability in signal interpretation, and surgeon expertise.

Median 8 (i.q.r. 1)

Outliers 2

96% rated 7–9

**Strong Consensus**

**Statement 16**: A persistent IONM amplitude below 100 µV following vagus nerve stimulation is highly predictive of postoperative vocal cord paralysis and should be considered a critical threshold for intraoperative decision-making. This presupposes an intact baseline signal (typically >500 µV) and requires that technical errors have been excluded.

Median 8 (i.q.r. 1)

Outliers 5

88% rated 7–9

**Strong Consensus**

**Statement 17**: The relative reduction in amplitude alone may not be a reliable predictor of postoperative vocal cord paralysis. A reduction of 50% from baseline amplitude, while maintaining an absolute amplitude above 100 µV, is associated with a very low risk of postoperative vocal cord paralysis. However, the threshold of a 50% persistent baseline reduction is not yet well documented in clinical studies and requires further investigation to establish its predictive value.

Median 8 (i.q.r. 1)

Outliers 5

88% rated 7–9

**Strong Consensus**

**Statement 18**: A marked reduction in IONM amplitude or LOS—relative to a stable baseline—should warrant an intraoperative assessment for nerve integrity, including dissection adjustments or staged thyroidectomy. Technical factors should be excluded.

Median 8 (i.q.r. 1)

Outliers 1

98% rated 7–9

**Very Strong Consensus**

**Statement 19**: In the event of LOS, the presence of a preserved laryngeal twitch may suggest intact neuromuscular function despite the monitoring failure. While this finding may support the hypothesis of a technical issue rather than true nerve injury, it should be interpreted with caution. The decision to proceed with contralateral surgery should not be based solely on the presence of a laryngeal twitch.

Median 8 (i.q.r. 1)

Outliers 4

92% rated 7–9

**Strong Consensus**

**Statement 20**: IONM reliability depends on proper set-up, user expertise, and adherence to standardized protocols. Most failures are preventable and rarely due to true equipment malfunction.

Median 8 (i.q.r. 1)

Outliers 1

98% rated 7–9

**Very Strong Consensus**

**Statement 21**: The interpretation of IONM signals requires standardized protocols to minimize inter-surgeon variability. Establishing uniform interpretation criteria is crucial for ensuring the reliability of IONM data and improving the comparability and validity of research findings.

Median 8 (i.q.r. 1)

Outliers 0

100% rated 7–9

**Very Strong Consensus**

In the Eurocrine^®^ registry encompassing 92 505 thyroid procedures with 137 849 nerves at risk, bilateral recurrent laryngeal nerve injury was documented in 66 nerves at risk (0.05%), while unilateral nerve injury was documented in 3804 nerves at risk (2.8%). These low rates were observed in a setting where IONM was used in 87.0% of procedures, underscoring the clinical relevance of reliable intraoperative signal preservation and structured intraoperative decision-making.

Survey data among ESES members further underline the perceived predictive value of IONM in daily practice. A large majority of respondents reported that IONM reliably predicts postoperative vocal cord function, with 62.7% stating that this is always the case and an additional 33.5% reporting that it is often reliable. Only a small minority considered IONM to have limited predictive value, reflecting broad clinical confidence in signal stability as a surrogate marker for nerve integrity. However, both registry data and survey responses highlight that predictive accuracy is influenced by how signals are interpreted. Definitions of LOS varied considerably among surgeons; 47.9% defined LOS as an EMG amplitude below 100 µV, 6.1% used a threshold below 200 µV, and 28.2% relied primarily on the absence of acoustic feedback. In addition, 63.8% of respondents reported that a reduction of more than 50% in vagus nerve amplitude influenced intraoperative decision-making, whereas 36.2% did not alter their strategy based on this finding alone. Findings from the literature indicate that preserved absolute EMG amplitudes, most commonly using thresholds around 100 µV, are frequently used as indicators of intact nerve function, whereas relative amplitude reductions alone are less consistently associated with postoperative vocal cord outcomes. However, these thresholds are not uniformly defined or validated across studies^13,14^.

Evidence on the clinical usefulness of a laryngeal twitch is limited and inconsistently reported. In the literature review, a laryngeal twitch was described as an additional criterion in 37 studies (17.8%), representing 11 845 patients, whereas 171 studies (representing 375 625 patients) provided no information on its use. Although a preserved laryngeal twitch may suggest intact neuromuscular function when EMG is absent or abnormal, the limited and heterogeneous reporting precludes firm conclusions on predictive value. Therefore, a laryngeal twitch should be considered a supplementary finding and should not be used alone to guide critical intraoperative decisions, including whether to proceed with contralateral surgery after LOS.

Overall, Eurocrine^®^ registry outcomes and survey data are consistent with the consensus view that IONM is a valuable predictor of postoperative vocal cord function, while also highlighting that its reliability depends on standardized protocols, clear signal definitions, and surgeon expertise in interpretation.

**Statement 22**: IONM findings should directly guide intraoperative decision-making to prevent bilateral recurrent laryngeal nerve injury.

Median 9 (i.q.r. 1)

Outliers 2

96% rated 7–9

**Very Strong Consensus**

**Statement 23**: The routine use of IONM decreases the incidence of bilateral vocal cord palsy only if the results of IONM prompt an intraoperative change of strategy.

Median 9 (i.q.r. 1)

Outliers 1

98% rated 7–9%

**Very Strong Consensus**

**Statement 24**: In the event of intraoperative recurrent laryngeal nerve injury indicated by IONM on the first side during thyroidectomy, a staged approach should be considered to avoid the risk of bilateral vocal cord palsy. The decision to proceed with contralateral surgery should take into account the underlying disease, the urgency of treatment, the likelihood of nerve recovery, and the patient’s preferences and values. In most cases, delaying completion surgery until nerve function is reassessed is the safer approach.

Median 8.5 (i.q.r. 1)

Outliers 1

98% rated % 7–9

**Very Strong Consensus**

In the literature, registry data, and survey responses, IONM is consistently used as a decision-support tool to reduce the risk of bilateral recurrent laryngeal nerve injury by guiding intraoperative strategy. In the systematic literature review, including 387 470 patients in 208 studies, a change in surgical strategy after LOS or abnormal IONM findings was explicitly described in 88 studies, representing 84 909 patients. Where reported, conversion to a staged thyroidectomy after intraoperative nerve injury on the first side was performed in 21.9% of patients, indicating that IONM findings were actively used to modify surgical plans.

Eurocrine^®^ registry data provide real-world confirmation of this practice. Among 45 052 planned total thyroidectomies, a change in surgical strategy after intraoperative nerve injury or signal alteration was documented for 1143 patients (2.5%), most commonly by converting the planned bilateral procedure to a staged approach. In this high-uptake setting for IONM (used in 87.0% of procedures), bilateral recurrent laryngeal nerve injury remained rare, occurring in 66 of 137 849 nerves at risk (0.05%), supporting the clinical relevance of strategy modification in response to intraoperative findings.

Survey data further illustrate the significant role of IONM in intraoperative decision-making. In the context of planned bilateral thyroid surgery, loss of IONM signal on the first side always led to a change in surgical strategy for 61.5% of respondents and often did so for 19.9% of respondents, while only a small minority of respondents reported rarely or never altering their approach. These findings align with the very strong consensus that routine use of IONM can reduce the risk of bilateral vocal cord palsy only when abnormal findings prompt an intraoperative change of strategy. After suspected intraoperative recurrent laryngeal nerve injury on the first side, a staged surgical approach should be considered as part of an individualized risk assessment. The decision to proceed with contralateral surgery should consider the underlying disease, urgency of treatment, likelihood of nerve recovery, and the patient’s preferences and values. In most cases, postponing completion surgery until postoperative reassessment of nerve function represents the safer option to minimize the risk of bilateral vocal cord palsy.

The full set of consensus statements is provided in *Table S1*.

## Discussion

This consensus statement addresses the gap between the widespread clinical use of IONM and the substantial heterogeneity in its documentation, interpretation, and reporting in the literature. By combining a systematic literature review, analysis of Eurocrine^®^ registry data, a survey among ESES members, and a structured Delphi consensus process, the present work defines key requirements for standardization in IONM research. The findings highlight that consistent application of monitoring algorithms, clear signal definitions, and standardized outcome assessment are essential to improve comparability and reproducibility across studies. The evidence base informing the present consensus is substantially stronger for thyroid surgery than for parathyroid surgery. Although several principles are likely applicable across endocrine neck surgery, specific data regarding parathyroid surgery remain limited, underlining an important gap for future research.

Over the past two decades, based on experimental investigations, clinical studies, and accumulated surgical experience, fundamental standards for IONM have been established and are now widely accepted. In cases of clear LOS (defined as an amplitude of 100 µV or less)^4^ or preserved stable signals throughout resection, postoperative vocal cord function can be predicted with a high degree of certainty. Relevant challenges, however, remain, particularly in the intraoperative interpretation of relative amplitude decline without complete LOS. In such intermediate scenarios, the probability of postoperative dysfunction may be difficult to estimate in individual patients, lying between the extremes of unequivocal signal loss and completely unchanged signals after resection. The effect of IONM on the prevention of transient and permanent recurrent laryngeal nerve injury remains variably reported. While some studies and meta-analyses suggest a potential benefit, particularly in high-risk procedures, the overall evidence is heterogeneous. RCTs are limited and often underpowered for rare outcomes such as permanent nerve palsy. Consequently, the present consensus does not aim to re-establish the general indication for IONM, but rather to improve standardization, documentation, and data quality to enable more robust evaluation in future studies.

Further refinement of predictive accuracy requires high-quality, methodologically transparent studies. To allow meaningful interpretation and comparison, future IONM studies should provide detailed reporting of neuromonitoring methodology and data integrity. This includes verification of raw neuromonitoring data quality and appropriate signal labelling (including signal validation by displayed EMG waveforms). Although amplitude-based criteria are most frequently reported, latency changes are also clinically relevant, particularly in progressive traction injury and continuous monitoring. Latency is, however, inconsistently reported in the literature, which limits its integration into current evidence summaries. Key technical parameters should be explicitly stated, including confirmation of an adequate initial EMG amplitude (≥500 µV) and correct electrode positioning before dissection, stimulation current, stimulation mode (intermittent or continuous), the method used to define latency (onset or peak), device settings and software version where relevant, type of stimulation probe (monopolar or bipolar), type of recording electrodes (integrated endotracheal tube electrodes, adhesive surface electrodes or needle electrodes), and standardized timing and anatomical locations of stimulation (for example V1–R1–R2–V2).

Equally important are clear and reproducible definitions. Studies should explicitly define how LOS was defined. It should also be specified how a ‘normal’ post-resection signal was defined and which criteria were used to assume preserved nerve function, including the amplitude threshold used (for example <100 µV, <200–250 µV, or relative decline from baseline such as >50% of V1).

Furthermore, the term ‘change of strategy’ must be precisely described, distinguishing between conversion from planned total thyroidectomy to hemi-thyroidectomy, temporary cessation of contralateral resection, and other intraoperative modifications in response to signal changes.

Finally, outcome reporting must include objective assessment of vocal cord function, preferably with standardized preoperative and postoperative laryngoscopy and documentation of the duration of any transient paresis. Only through consistent methodological transparency across these domains can the predictive value of IONM be further refined, thereby substantially improving inter-study comparability.

As with any consensus initiative informed by the literature, registry data, and survey responses, the present statements should be interpreted within their methodological context. The underlying literature is predominantly retrospective and monocentric, registry data lack detailed intraoperative algorithmic information, and survey findings reflect practices among surgeons with a particular interest in IONM. Accordingly, the statements represent structured expert agreement rather than causal proof.

Previous recommendations on IONM have been published by other groups, most notably the International Neuromonitoring Study Group^3,4^. While these have established important technical and procedural standards, the present consensus expands on these by placing greater emphasis on data quality, standardized reporting, and research requirements. This reflects the increasing need for comparability across studies and the integration of registry-based data in contemporary endocrine surgery research.

This consensus statement provides a structured framework for the standardized use and reporting of IONM in thyroid surgery. By integrating evidence from the literature, registry data, and expert consensus, it aims to improve data quality, comparability, and clinical interpretation. Future studies should build on these standards to strengthen the evidence base and further refine the role of IONM in clinical practice.

## Supplementary Material

znag090_Supplementary_Data

## Data Availability

Data underlying this article are derived from published literature, survey data, and the Eurocrine^®^ registry. Access to Eurocrine^®^ registry data is subject to governance and may be available upon reasonable request and appropriate approvals.
