# Supplementary material for: European Society of Endocrine Surgeons (ESES) consensus statement on standardization and research requirements for intraoperative nerve monitoring in endocrine surgery
Source: Br J Surg. 2026 Aug 6;113(8):znag090. doi: 10.1093/bjs/znag090 (PMC13446503; doi:10.1093/bjs/znag090)
Supplement: Supplementary file 1 [file znag090_supplementary_data.docx]

**European Society of Endocrine Surgeons (ESES) Consensus Statement on Standardization and Research Requirements for Intraoperative Nerve Monitoring in Endocrine Surgery**

Katrin Brauckhoff^1,2^, Agata Dukaczewska^3^, Pietro Princi^4,5^, Rick Schneider^6^, Sam Van Slycke^7^, Selen Soylu Yaliman^8^, Julia I. Staubitz-Vernazza^9^, Neil S. Tolley^10^, Kyriakos Vamvakidis^11^, Ilias A. Zorbas^12^, Thomas J. Musholt^13^

**Affiliations**

1. Department of Breast and Endocrine Surgery, Haukeland University Hospital, Bergen, Norway, katrin.brauckhoff@helse-bergen.no

2. Department of Clinical Science, University of Bergen, Bergen, Norway

3. Digital Health Cluster, Hasso-Plattner Institute, Potsdam, Germany, a_dukaczewska@icloud.com

4. Multifunctional Center of Endocrine Surgery, Cristo Re General Hospital, Rome, Italy

5. Unicamillus International Medical University, Rome, Italy, pietro.princi@unicamillus.org

6. Department of Visceral, Vascular and Endocrine Surgery, Martin Luther University of Halle-Wittenberg, Halle (Saale), Germany, rick.schneider@uk-halle.de

7. Department of General and Endocrine Surgery, AZORG, Aalst, Belgium, sam.van.slycke@azorg.be

8. Department of General Surgery, Istanbul University-Cerrahpasa, Cerrahpasa Faculty of Medicine, Istanbul, Turkey, selen_soylu@yahoo.com

9. Section of Endocrine Surgery, Department of General, Visceral and Transplantation Surgery, University Medical Center of the Johannes Gutenberg University Mainz, Mainz, Germany, julia.i.staubitz@gmail.com

10. Department of Endocrine and Thyroid Surgery, Hammersmith Hospital, Imperial College NHS Healthcare Trust, London, UK, Neil.Tolley@nhs.net

11. Department of Endocrine Surgery, Henry Dunant Hospital Center, Athens, Greece, [vamkyr@yahoo.com](mailto:vamkyr@yahoo.com)

12. Department of Endocrine Surgery, Henry Dunant Hospital Center, Athens, Greece, zorbashlias@gmail.com

13. Section of Endocrine Surgery, Department of General, Visceral and Transplantation Surgery, University Medical Center of the Johannes Gutenberg University Mainz, Mainz, Germany, [musholt@uni-mainz.de](mailto:musholt@uni-mainz.de)

**Corresponding author:** Katrin Brauckhoff, katrin.brauckhoff@helse-bergen.no **ORCID ID**: 0000-0002-2442-3281

| **Supplementary Figures and Tables** |  |
| --- | --- |
| **Table S1.** ESES consensus statements on intraoperative nerve monitoring (IONM) in endocrine surgery. Statements were developed through a structured Delphi process and rated on a 9-point Likert scale. Consensus was defined as a median score of 7–9 with an interquartile range (IQR) ≤2 and ≥75% of responses within the 7–9 range. |  |

| Statement no. | Domain | Summary statement | Median (IQR) | % rated 7–9 | Consensus level |
| --- | --- | --- | --- | --- | --- |
| 1 | General use | IONM supports nerve identification and intraoperative decision-making | 9 (1) | 96 | Strong |
| 2 | General use | IONM improves intraoperative decision making | 9 (1) | 98 | Very strong |
| 3 | General use | IONM should be available in high-risk and redo surgery | 9 (0) | 100 | Very strong |
| 4 | General use | Use of IONM should be considered as standard care | 8 (1) | 92 | Strong |
| 5 | Stimulation algorithm | Pre- and post-dissection vagus stimulation is essential for baseline assessment | 9 (1) | 98 | Very strong |
| 6 | Technical aspects | IONM systems provide equivalent functionality, but differ in data storage and documentation methods | 8 (1) | 94 | Strong |
| 7 | Stimulation parameters | Standard stimulation settings are comparable; awareness of supramaximal stimulation is required | 8 (1.25) | 85 | Strong |
| 8 | Recording electrodes | Electrode type and placement influence signal quality and interpretation | 8 (1) | 98 | Strong |
| 9 | Preoperative assessment | Preoperative laryngoscopy is recommended in patients with risk factors | - | 98 | After  voting |
| 10 | Preoperative assessment | Routine preoperative laryngoscopy detects asymptomatic palsy and is essential for research baseline assessment | - | 81 | After  voting |
| 11 | Postoperative assessment | Postoperative laryngoscopy should be performed when nerve injury is suspected | - | 97 | After voting |
| 12 | Postoperative  assessment | Objective laryngoscopy is required for outcome validation in research | 9 (1) | 90 | Strong |
| 13 | Vocal cord assessment | Laryngoscopy remains the gold standard for vocal cord assessment | 9 (1) | 94 | Strong |
| 14 | Prediction | Stable IONM signals correlate with preserved vocal cord function | 8 (1) | 94 | Strong |
| 15 | Prediction | Predictive accuracy depends on standardized protocols and interpretation | 8 (1) | 96 | Strong |
| 16 | Signal interpretation | LOS definition must be clearly defined and standardized | 8 (1) | 88 | Strong |
| 17 | Signal interpretation | Relative amplitude reduction alone is not predictive | 8 (1) | 88 | Strong |
| 18 | Signal  interpretation | LOS or amplitude reduction requires intraoperative assessment | 8 (1) | 98 | Very strong |
| 19 | Signal interpretation | Laryngeal twitch is supplementary and not sufficient alone for decision-making | 8 (1) | 92 | Strong |
| 20 | Intraoperative management | IONM reliability depends on setup and expertise; most failures are preventable | 8 (1) | 98 | Very strong |
| 21 | Intraoperative  management | Standardized protocols are required for reliable and comparable IONM signal interpretation | 8 (1) | 100 | Very strong |
| 22 | Surgical strategy | IONM should guide decisions to prevent bilateral nerve injury | 9 (1) | 96 | Very strong |
| 23 | Surgical strategy | IONM reduces bilateral nerve injury only findings prompt change of strategy | 9 (1) | 98 | Very strong |
| 24 | Surgical  strategy | Staged surgery should be considered after first -side nerve injury | 8.5 (1) | 98 | Very strong |

| **Table S2.** Technical characteristics of three commonly used intraoperative nerve monitoring (IONM) systems. All systems use constant current stimulation and provide comparable core functionality for nerve stimulation and EMG recording. Differences relate mainly to stimulation settings, electrode configuration, data storage and export. |  |
| --- | --- |
|  |  |
|  |  |

| Parameter | Dr.Langer Medical | Inomed | Medtronic | Comments |
| --- | --- | --- | --- | --- |
| System details |  |  |  |  |
| System | Avalanche® SI2 | C2 Xplore® | NIM Vital™ |  |
| Operating system | Microsoft Windows 10 | Microsoft Windows 10 | Microsoft Windows 10 |  |
| Stimulation type | Constant current | Constant current | Constant current |  |
| Current range (mA) | 0.05–10 | 0.01–25 | 0.01–50 | Surgeon adjustable |
| Frequency range (Hz) | 3–4 | 0.1–60 | 1–20 |  |
| Help menu | No | Yes | Yes |  |
| Integrated data storage | Yes | Yes | No |  |
| HL7 integration | Yes | Yes | No | Hospital integration possible |
| USB connection | Yes | Yes | Yes |  |
| Stimulation devices – intermittent IONM |  |  |  |  |
| Monopolar | Yes | Yes | Yes |  |
| Standard control | Yes | Yes | Yes |  |
| Surgeon-controlled probe | No | No | Yes |  |
| Long probe (minimally invasive) | Yes | Yes | Yes |  |
| Bipolar | Yes | Yes | Yes |  |
| Continuous IONM (C-IONM) |  |  |  |  |
| Electrode type | Saxophone | Delta | APS |  |
| Monopolar | No | No | Yes |  |
| Bipolar | Yes | Yes | No |  |
| Different device sizes | No | No | Yes |  |
| Stimulation settings – intermittent IONM |  |  |  |  |
| Frequency (Hz) | 4 | 3 | 4 |  |
| Pulse width (µs) | 200 | 200 | 100 |  |
| Current (mA) | 1 | 1 | 1 | Adjustable |
| Stimulation settings – C-IONM |  |  |  |  |
| Frequency (Hz) | 3 | 1 | 1 |  |
| Pulse width (µs) | 200 | 200 | 100 |  |
| Current (mA) | 1 | 1 | 1 | Adjustable |
| Baseline definition | User-defined | Measured at baseline | Averaged from 20 samples |  |
| Artefact suppression | Yes | Yes | Yes | Adjustable |
| Recording devices |  |  |  |  |
| EMG tube | Adhesive or flexible electrodes | Standard or adhesive electrodes | Integrated EMG tube |  |
| Number of recording channels | 1 | 4 | 1–2 |  |
| Needle electrodes | Compatible | Yes | Yes |  |
| EMG calculation |  |  |  |  |
| Amplitude | Peak-to-peak | Peak-to-peak | Peak-to-peak |  |
| Latency | Onset latency | Onset latency | Onset latency |  |
| Onset latency default | Yes | Yes | Yes |  |
| Peak latency selectable | No | No | Yes |  |
| Display and monitoring |  |  |  |  |
| Number of displayed channels | 1 | 4 | 1–2 |  |
| Channel selection | N/A | Selectable | Highest amplitude |  |
| EMG events (AMP/LAT) | Yes | Yes | Yes |  |
| Nerve trend | No | Yes | Yes |  |
| EMG display (C-IONM) | Yes | Yes | Yes |  |
| Trend display (AMP/LAT) | Yes | Yes | Yes |  |
| Latency threshold (%) | +10 | +10 | +10 | Adjustable |
| Amplitude threshold (%) | −50 | −50 | −50 | Adjustable |
| Data storage and documentation |  |  |  |  |
| EMG waveform (intermittent) | Snapshots + database | Snapshots + database | Snapshots | INMSG standard |
| EMG waveform (C-IONM) | Snapshots + database | Yes | Snapshots |  |
| Continuous EMG storage | Yes | Yes | No |  |
| Trend reports | Yes | Yes | Yes |  |
| Scientific data export |  |  |  |  |
| Intermittent export | Raw signals | Raw + CSV | Tagged reports |  |
| C-IONM export | TDMS (Excel compatible) | Proprietary + CSV | Database export |  |

**Supplementary Results**
